# Association Between Herpes Zoster and Risk of Incident Fragility Fractures in US Veterans: A Matched Cohort Study

**DOI:** 10.1111/jgs.70174

**Published:** 2025-10-17

**Authors:** Calif A. A. Yousuf, Julie A. Womack, Roger J. Bedimo, Christopher T. Rentsch, Charlotte Warren‐Gash

**Affiliations:** ^1^ Faculty of Epidemiology & Population Health London School of Hygiene and Tropical Medicine London UK; ^2^ College of Life Sciences Leicester Medical School Leicester UK; ^3^ VA Connecticut Healthcare System West Haven Connecticut USA; ^4^ Yale School of Nursing West Haven Connecticut USA; ^5^ VA North Texas Health Care System, UT Southwestern Dallas Texas USA; ^6^ Department of Internal Medicine Yale School of Medicine New Haven Connecticut USA

**Keywords:** electronic health records, fragility fracture, herpes zoster, osteoporosis, veterans

## Abstract

**Background:**

Herpes zoster (HZ) and fragility fractures typically affect older adults and present major burdens to healthcare systems. While HZ is associated with an increased risk of neurological, ocular, skin, and visceral complications, it is unclear whether it affects long‐term bone health. Therefore, we aimed to compare the risk of fragility fractures in Veterans with HZ relative to matched Veterans without HZ in the United States.

**Methods:**

We used routinely collected data from the Veterans Aging Cohort Study (VACS‐National) from 01/01/2008 to 31/12/2023. Veterans with incident HZ diagnoses aged 40+ were matched on age, sex, race, ethnicity, site of care, and calendar time with up to five Veterans without HZ. The association between HZ and subsequent fragility fractures (defined as hip/femoral, shoulder/upper arm, vertebral, and wrist/forearm fractures) overall and by site was assessed using a Cox model stratified by matched set adjusting for sociodemographic, lifestyle, and clinical confounders. Age, frailty, and receipt of antivirals (AVT) within 7 days of exposure were investigated as potential effect modifiers for the relationship.

**Results:**

We included 229,992 Veterans with HZ matched to 1,134,519 Veterans without HZ. Both groups were comparable in median age (67.8 vs. 67.7 years), percentage males (93.5% vs. 93.7%), and median follow‐up time (5.9 vs. 5.4 years). Veterans with HZ had a 15% higher risk of incident fragility fracture (aHR = 1.15, 95% CI: 1.13, 1.18) compared to Veterans without HZ. The increased risk was observed for each fracture site. There was evidence of interaction by age, frailty, and receipt of AVT, as older, frailer, and AVT‐treated Veterans had a higher risk of fragility fracture.

**Conclusions:**

Veterans with HZ were at a higher risk of fragility fractures relative to Veterans without HZ, highlighting the need for improved fracture prevention among those diagnosed with HZ. Further research in non‐Veteran and female populations will improve generalizability.

## Graphical abstract

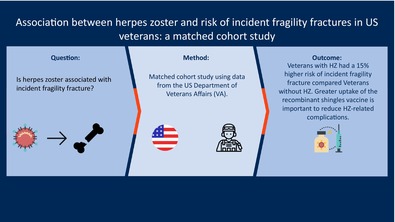

**Summary**

- Key points
  ○ Herpes zoster was associated with an increased risk of fragility fracture in US Veterans.
  ○ The association was modified by age, level of frailty, and receipt of antivirals (AVT) within 7 days of exposure.
- Why does this paper matter?
  ○ This large national cohort study provides evidence that individuals diagnosed with herpes zoster had a 15% higher risk of developing fragility fractures compared to those without the diagnosis, addressing a gap in previously limited evidence. It highlights the importance of greater uptake of the HZ vaccine in eligible adults.

## 1 Introduction

Herpes zoster (HZ) is caused by the reactivation of the varicella‐zoster virus (VZV), the same virus responsible for chickenpox. HZ is responsible for a significant public health burden, particularly in aging populations [1]. The primary risk factor for HZ is increasing age, due to a natural decline in cellular immunity to the VZV [2]. In the United States, one in three will develop shingles in their lifetime, and there are an estimated 1 million cases annually [3, 4]. HZ may be associated with post‐herpetic neuralgia as well as other neurological, ocular, skin, and visceral complications [5]. It can be effectively prevented by receiving two doses of the recombinant zoster vaccine regardless of history of prior episodes [6].

Fragility fractures are defined as “fractures most likely to occur from low‐energy trauma such as a fall from standing height or less” [7]. They are associated with osteoporosis, which is a condition characterized by low bone mineral density (BMD) and deterioration of bone tissue structure [8]. Common sites for fragility fractures include the vertebrae, proximal femur, wrist, and proximal humerus [9]. Chronic inflammatory conditions such as inflammatory bowel disease and rheumatoid arthritis are linked to an increased osteoporosis risk [10, 11]. However, it is less clear whether transient inflammatory episodes, such as those occurring with HZ, have long‐term effects on bone health.

Three studies using claims data from Taiwan and Korea have suggested associations between HZ and osteoporosis risk [12, 13, 14]. A single matched cohort study using data from the Taiwanese National Health Insurance Research Database found that patients with HZ had increased fracture risks ranging from 10% to 38% depending on the site compared to patients without HZ [12]. However, the study was not able to adjust for key confounders such as body mass index (BMI), alcohol, or smoking, or to explore whether receiving treatment with AVT modified the risk of fracture after HZ. No large health data studies have been conducted in other geographic settings.

We therefore aimed to investigate the association between HZ and the risk of incident fragility fractures in the United States using data from the Veterans Aging Cohort Study–National Cohort (VACS‐National). We also aimed to explore the association of HZ on each type of fragility fracture (hip/femoral, shoulder/upper arm, vertebral, and wrist/forearm) individually. Lastly, we aimed to identify whether the association between HZ and any incident fragility fracture is modified by age, frailty, or receipt of antiviral within 7 days of exposure.

## 2 Methods

### 2.1 Data Source

The VACS‐National represents all Veterans (> 14.1 million) who have accessed the US Department of Veterans Affairs (VA) care since 2000. The VA is the largest integrated healthcare system in the United States, providing care to over 9 million Veterans each year at over 1300 health care facilities (including 172 VA Medical Centres and 1138 outpatient sites). All care within the VA is documented in a rich, national resource of highly detailed EHR data. There are > 100 variables cleaned and ready for analysis in VACS‐National, including demographics, comorbidities, pharmacy dispensing records, labs, vital signs, and other lifestyle factors [15]. Diagnoses are recorded using the International Classification of Diseases, 9th (ICD‐9) and 10th revision (ICD‐10) [16, 17].

### 2.2 Study Design and Population

**FIGURE 1:**
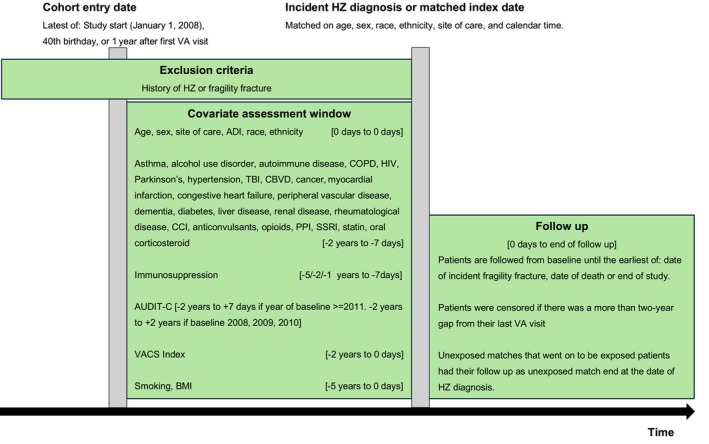
Illustration of study design.

We conducted a matched cohort study to compare the risk of incident fragility fractures among Veterans with a prior diagnosis of HZ to that of matched unexposed Veterans (Figure 1). The study period was between 1 January 2008 and 31 December 2023. Veterans entered the eligible cohort at the latest of: study start (1 January 2008), 40th birthday, or 1 year after their first VA visit. Veterans were excluded if they had a history of HZ or fragility fracture prior to cohort entry. Eligible Veterans with HZ were matched with up to five Veterans without a diagnosis of HZ on age (within 365 days), sex, race, ethnicity, site of care, and calendar time (Figure S1).

### 2.3 Follow Up

The index date for Veterans with HZ was defined as the date of incident HZ diagnosis. For Veterans without HZ, the index date is defined as the date of incident HZ in their HZ match. Veterans were followed from the index date until the earliest of: incident fragility fracture, death, end of study, or 2 years from last VA visit. Follow‐up for Veterans without HZ was also censored at the date of incident HZ. The VA advises Veterans in their care to have health visits roughly annually and therefore Veterans were censored if there was more than a 2‐year gap since their last VA visit to ensure that Veterans under follow‐up had equal opportunity for outcome assessment. A patient initially identified as not having HZ will later enter the study as an HZ patient if they developed incident HZ.

### 2.4 Exposure, Outcome and Covariates

The exposure was incident HZ defined as at least one inpatient or outpatient diagnosis of HZ or acute HZ complications using ICD‐9 and ICD‐10 codes [16, 17] (Table S1). Diagnostic codes related only to chronic complications were excluded as these codes do not clearly indicate the onset date for HZ episodes. The codes used for identifying HZ have been used and validated in prior research [18, 19]. To explore effect modification by AVT, a three‐level exposure variable was created that stratified the HZ group by receipt of AVT within seven days of HZ diagnosis (Unexposed to HZ, HZ without AVT, and HZ with AVT).

The outcome was incident fragility fracture, which was defined as “fractures most likely to occur from low‐energy trauma such as a fall from standing height or less” [7]. The fracture sites included were hip/femoral, shoulder/upper arm, vertebral, and wrist/forearm. Fractures were identified using ICD‐9 and ICD‐10 codes that have been previously validated in VA data [7] (Table S2).

All potential confounders were identified a priori based on clinical reasoning, prior evidence exploring this potential association, and a directed acyclic graph (Figure S2). Covariates included asthma, autoimmune disease, cancer and/or metastases, myocardial infarction, congestive heart failure, peripheral vascular disease, cerebrovascular disease, chronic obstructive pulmonary disease, dementia, diabetes, human immunodeficiency virus, immunosuppression, hypertension, liver disease, Parkinson's disease, rheumatological disease, anticonvulsants, oral corticosteroids, prescription opioids, proton pump inhibitors, statins, selective serotonin reuptake inhibitors, Area Deprivation Index (ADI), alcohol status, BMI, smoking status, Charlson Comorbidity Index (CCI), and VACS Index (version 2.0). Briefly, the CCI is a measure of overall comorbidity burden and is based on diagnostic codes across 17 clinical domains [20]. The VACS Index is a validated and generalizable measure of physiologic frailty and is based on routinely available laboratory measurements [21, 22]. Codelists for all clinical comorbidities are available in this data repository (https://datacompass.lshtm.ac.uk/id/eprint/2848/) and in Table 1 of a prior publication (https://pubmed.ncbi.nlm.nih.gov/16224307/) [20, 23]. Further details on covariates and their handling are provided in Text S1.

**TABLE 1:** Baseline characteristics by HZ status.

Characteristic	Veterans without HZ, *N* = (1,134,519)	Veterans with HZ, *N* = (229,992)
Age at baseline^a^		
Median (IQR)	67.7 (61.4–75.7)	67.8 (61.4–75.8)
Sex, *n* (%)^a^		
Male	1,062,648 (93.7)	214,958 (93.5)
Race and ethnicity, *n* (%)^a^		
White	841,251 (74.2)	170,179 (74.2)
Black	146,438 (12.9)	29,550 (12.8)
Hispanic	66,703 (5.9)	13,719 (6.0)
Asian	6410 (0.6)	1414 (0.6)
American Indian/Alaska Native	6290 (0.6)	1319 (0.6)
Native Hawaiian/Pacific Islander	6999 (0.6)	1439 (0.6)
Mixed race	6625 (0.6)	1460 (0.6)
Missing	53,803 (4.7)	10,912 (4.7)
Baseline year, *n* (%)^a^		
2008–2011	359,928 (31.7)	72,510 (31.5)
2012–2015	402,079 (35.4)	82,470 (35.9)
2016–2019	248,811 (21.9)	50,045 (21.7)
2020–2022	123,701 (10.9)	24,967 (10.8)
Years of follow up		
Median (IQR)	5.4 (2.5–8.9)	5.9 (2.8–9.4)
Area Deprivation Index, *n* (%)		
Quintile 1—Least deprived	226,088 (20.0)	44,789 (19.5)
Quintile 2	231,078 (20.4)	45,452 (19.8)
Quintile 3	220,685 (19.5)	44,658 (19.4)
Quintile 4	223,333 (19.7)	46,130 (20.0)
Quintile 5—Most deprived	209,852 (18.5)	43,655 (19.0)
Missing	23,483 (2.1)	5308 (2.3)
Body mass index category, *n* (%)		
Underweight (< 18.5 kg/m^2^)	8416 (0.7)	2245 (1.0)
Normal (18.5 kg/m^2^–24.9 kg/m^2^)	183,428 (16.2)	44,852 (19.5)
Overweight (25.0 kg/m^2^–29.9 kg/m^2^)	357,068 (31.5)	83,085 (36.1)
Obese (> 30 kg/m^2^)	398,391 (35.1)	90,444 (39.3)
Missing	187,216 (16.5)	9366 (4.1)
Smoking status, *n* (%)		
Never	345,400 (30.4)	72,531 (31.5)
Former	414,586 (36.5)	89,043 (38.7)
Current	325,666 (28.7)	65,849 (28.6)
Missing	48,867 (4.3)	2569 (1.1)
Alcohol use, *n* (%)		
Abstinent without AUD	487,363 (43.0)	117,807 (51.2)
Abstinent with AUD	17,701 (1.6)	5106 (2.2)
Lower‐risk consumption	325,619 (28.7)	70,967 (30.9)
Moderate‐risk consumption	95,707 (8.4)	18,828 (8.2)
High‐risk consumption or AUD	49,667 (4.4)	10,722 (4.7)
Missing	158,462 (14.0)	6562 (2.9)
VACS Index score (quartiles)^b^		
Quartile 1 (< 62.0)—Least frail	201,281 (17.7)	48,558 (21.1)
Quartile 2 (≥ 62.0 to < 69.8)	202,615 (17.9)	46,445 (20.2)
Quartile 3 (≥ 69.8 to < 79.1)	202,640 (17.9)	47,226 (20.5)
Quartile 4 (≥ 79.1)—Most frail	195,778 (17.3)	53,748 (23.4)
Missing	332,205 (29.3)	34,015 (14.8)
Charlson Comorbidity Index		
Mean (SD)	1.3 (1.7)	1.9 (2.1)
Baseline comorbidity, *n* (%)		
Asthma	30,048 (2.6)	8511 (3.7)
Autoimmune disease	45,129 (4.0)	15,849 (6.9)
Cancer	123,884 (10.9)	36,544 (15.9)
Myocardial infarction	30,197 (2.7)	9586 (4.2)
Congestive heart failure	69,135 (6.1)	22,151 (9.6)
Peripheral vascular disease	90,867 (8.0)	25,399 (11.0)
Cerebrovascular disease	82,120 (7.2)	22,704 (9.9)
COPD	198,563 (17.6)	56,133 (24.4)
Dementia	18,414 (1.6)	4613 (2.0)
Diabetes	326,030 (28.7)	77,909 (33.9)
HIV	3638 (0.3)	2348 (1.0)
Hypertension	610,661 (53.8)	141,933 (61.7)
Immunosuppression	25,685 (2.3)	13,422 (5.8)
Liver disease	47,949 (4.2)	12,994 (5.6)
Parkinson's	9491 (0.8)	1920 (0.8)
Renal disease	93,475 (8.2)	27,398 (11.9)
Rheumatological disease	17,558 (1.5)	6611 (2.9)
Dispensed medications, *n* (%)		
Anticonvulsants	183,893 (16.2)	53,726 (23.4)
Prescription opioid	304,826 (26.9)	93,453 (40.6)
Oral corticosteroid	100,167 (8.8)	40,962 (17.8)
PPI	319,557 (28.2)	89,900 (39.1)
SSRI	178,122 (15.7)	47,032 (20.4)
Statin	544,385 (48.0)	136,053 (59.2)

Abbreviations: AUD, alcohol use disorder; COPD, chronic obstructive pulmonary disease; HIV, human immunodeficiency virus; PPI, proton pump inhibitors; SSRI, selective serotonin reuptake inhibitors; VACS, Veterans Aging Cohort Study.

^a^ These variables have been accounted for in the matching process.

^b^ Excluded from the main analysis due to substantial missingness.

### 2.5 Statistical Analysis

We summarized the study variables by HZ diagnosis, presenting % for categorical variables or median (IQR) for continuous variables, with missingness reported where relevant. We calculated the crude incidence rate (per 1000 person‐years) of all fragility fractures by HZ diagnosis. We also conducted descriptive statistics on the time to fracture (median, IQR) overall and for each specific type of fragility fracture by HZ status. An unadjusted cumulative incidence function plot was generated to produce a visual representation of time to fragility fracture by HZ status whilst appropriately accounting for the competing risk of death.

We utilized a Cox proportional hazards model to estimate the association between HZ and all fragility fractures using age as the underlying timescale. Each model was stratified by matched set and thus implicitly adjusted for the matching variables. Our fully adjusted models additionally adjusted for the covariates specified above. We then estimated the association between HZ and each type of fragility fracture (wrist/forearm, hip/femoral, vertebral, and shoulder/upper arm). A complete case analysis was conducted, and this is considered a valid approach where missingness has been determined to be missing not at random (Text S2) [24]. Due to substantial missingness, VACS Index was excluded from our main analyses, but we additionally adjusted for it to control for physiologic frailty in a sensitivity analysis [21].

We explored effect modification by age (in 10‐year age bands), physiologic frailty (measured by quartiles of VACS Index), and receipt of AVT within seven days of HZ diagnosis. To assess effect modification by age and physiologic frailty, we presented stratum‐specific hazard ratios, 95% CIs, and *p* values from the likelihood‐ratio test comparing the model with and without the interaction term. To assess effect modification by receipt of AVT, we presented stratum‐specific hazard ratios (no HZ diagnosis (reference group), HZ without AVT, and HZ with AVT), 95% CIs, and *p* values from the likelihood‐ratio test comparing the model with the 3‐level HZ exposure to the model with the 2‐level HZ exposure.

Additional detail on data management and statistical analysis can be found in Text S3.

### 2.6 Sensitivity Analysis

Our first sensitivity analysis restricted the study population to those with index dates earlier than 31 December 2019 to reduce the risk of exposure misclassification due to potential changes in health‐seeking behavior during the COVID‐19 pandemic. Our second sensitivity analysis included variables with greater than 20% missingness (VACS Index) to reduce the risk of confounding by those variables as compared to the main model in which they were excluded. All data management and statistical analyses were conducted using Stata Version 18 (StataCorp. 2023).

### 2.7 Approvals and Reporting Guidelines

VACS‐National has been approved by the Institutional Review Boards of the London School of Hygiene and Tropical Medicine (30804), VA Connecticut Healthcare System (AJ0013), and Yale University (1506016006). It has been granted a waiver of informed consent and is Health Insurance Portability and Accountability Act compliant.

The study adhered to Strengthening the Reporting of Observational Studies in Epidemiology (STROBE) and Reporting of Studies Conducted Using Observational Routinely Collected Health Data (RECORD) guidelines (Table S3).

## 3 Results

### 3.1 Descriptive Analysis

**FIGURE 2:**
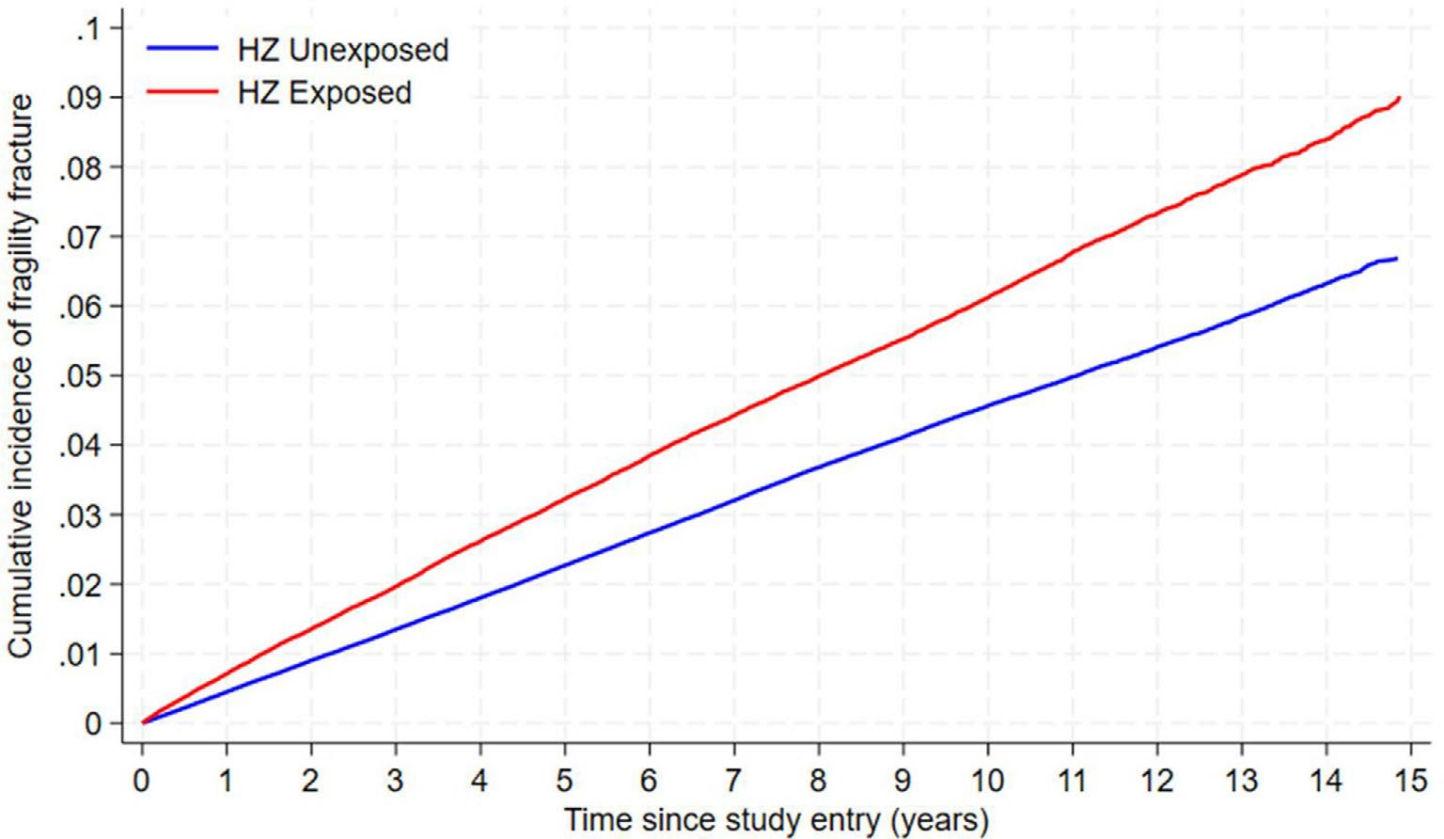
Cumulative incidence function of fragility fracture by herpes zoster exposure status.

As seen in Table 1 a total of 1,364,511 Veterans were successfully matched (229,992 Veterans with HZ and 1,134,519 Veterans without HZ). By design, the distribution of matching variables—age, sex, race, ethnicity, and baseline year—was similar across groups. The prevalence of all recorded clinical comorbidities was higher in Veterans with HZ compared to Veterans without HZ, and this is reflected in the higher mean for the CCI (*M* = 1.9, SD = 2.1 vs. *M* = 1.3, SD = 1.7). Furthermore, the dispensation of all included medication classes was also higher in Veterans with HZ compared to Veterans without HZ. The variables with missing data were: VACS Index (26.8%), BMI (14.4%), alcohol use (12.1%), smoking status (3.8%), and ADI (2.1%). The median time to fracture overall was 4 years (IQR: 1.8, 6.8) for Veterans without HZ and 4 years (IQR: 1.7, 6.9) for Veterans with HZ. Average times to each specific type of fragility fracture are reported in Text S4. The cumulative incidence estimates at the end of follow‐up were 9.0% in Veterans with HZ and 6.7% in Veterans without HZ (Figure 2).

### 3.2 Comparison of Incident Fragility Fractures in Veterans With HZ Compared to Matched Veterans Without HZ

During follow‐up, 10,499 Veterans with HZ and 33,932 Veterans without HZ experienced incident fragility fractures. The crude fragility fracture incidence rates were 7.29 (95% CI: 7.16, 7.44) and 5.09 (95% CI: 5.03, 5.14) per 1000 person‐years respectively (Table 2).

**TABLE 2:** Association between incident HZ and incident fragility fractures.

Herpes zoster diagnosis	*N* of patients^a^	*N* of fragility fracture^a^	Crude fragility fracture incidence rate per 1000 person years (95% CI)	Minimally adjusted Model HR (95% CI)	Fully adjusted model HR (95% CI)
Primary outcome					
All fractures					
No HZ	822,062	27,191	5.09 (5.03–5.14)	1 (reference)	1 (reference)
HZ	207,967	9736	7.29 (7.16–7.44)	1.42 (1.39–1.45)	1.15 (1.13–1.18)
Secondary outcomes					
Hip/femoral					
No HZ	822,062	5266	1.03 (1.00–1.05)	1 (reference)	1 (reference)
HZ	207,967	1863	1.41 (1.36–1.48)	1.33 (1.27–1.40)	1.08 (1.02–1.14)
Shoulder/upper arm					
No HZ	822,062	4136	0.76 (0.74–0.78)	1 (reference)	1 (reference)
HZ	207,967	1425	1.08 (1.03–1.13)	1.42 (1.35–1.50)	1.15 (1.09–1.22)
Vertebral					
No HZ	822,062	10,920	2.02 (1.99–2.06)	1 (reference)	1 (reference)
HZ	207,967	4015	3.00 (2.91–3.09)	1.47 (1.42–1.52)	1.18 (1.14–1.22)
Wrist/forearm					
No HZ	822,062	6869	1.27 (1.25–1.30)	1 (reference)	1 (reference)
HZ	207,967	2433	1.81 (1.74–1.88)	1.40 (1.35–1.46)	1.17 (1.12–1.22)

*Note*: Minimal models implicitly adjusted for matching factors: age, sex, race, ethnicity, site of care, and calendar time. Full models additionally adjusted for: asthma, autoimmune disease, cancer and/or metastases, myocardial infarction, congestive heart failure, peripheral vascular disease, cerebrovascular disease, chronic obstructive pulmonary disease, dementia, diabetes, human immunodeficiency virus, immunosuppression, hypertension, liver disease, Parkinson's disease, rheumatological disease, anticonvulsants, oral corticosteroids, prescription opioids, proton pump inhibitors, statins, selective serotonin reuptake inhibitors, Area Deprivation Index, alcohol status, body mass index, smoking status and Charlson Comorbidity Index.

^a^ In the fully adjusted model.

In the minimally adjusted model implicitly adjusting for matching factors, the HR for incident fragility fractures following HZ diagnosis in Veterans was 1.42 (95% CI: 1.39, 1.45). In the fully adjusted model, Veterans with HZ had a 15% higher risk (HR = 1.15, 95% CI: 1.13, 1.18) of incident fragility fracture compared to Veterans without HZ.

When stratifying the outcome by fracture type, the HR for the association between incident HZ and incident fragility fractures in the fully adjusted model was 1.18 (95% CI: 1.14, 1.22) for vertebral, 1.17 (95% CI: 1.12, 1.22) for wrist/forearm, 1.15 (95% CI: 1.09, 1.22) for shoulder/upper arm, and 1.08 (95% CI: 1.02, 1.14) for hip/femoral.

### 3.3 Effect Modification

There was evidence of effect modification by age (*p*‐interaction < 0.0001), with those aged over 80 experiencing the highest risk of fragility fracture after HZ (HR = 1.40, 95% CI: 1.32, 1.48) (Table 3).

**TABLE 3:** Association between incident HZ and incident fragility fractures stratified by age group, physiologic frailty and receipt of AVT.

Variable and levels	*N* of patients	*N* of fragility fractures	Fully adjusted model HR (95% CI)	*p*‐interaction^a^
Age group (years)				
40–49	55,241	1443	1.14 (1.02–1.27)	< 0.0001
50–59	152,719	5847	1.05 (0.99–1.12)	
60–69	406,680	15,257	1.07 (1.03–1.11)	
70–79	269,138	8574	1.23 (1.18–1.29)	
80+	146,251	5806	1.40 (1.32–1.48)	
	=1,030,029			
VACS Index (Quartiles)				
Quartile 1—Least frail	223,395	6421	1.11 (1.05–1.18)	=0.03
Quartile 2	221,172	7893	1.11 (1.05–1.17)	
Quartile 3	213,551	8290	1.15 (1.09–1.21)	
Quartile 4—Most frail	197,998	9203	1.25 (1.19–1.31)	
	=856,116			
HZ (3 Level exposure)				
Unexposed	822,062	27,191	1 (reference)	< 0.0001
Exposed without AVT	112,748	4735	1.04 (1.01–1.07)	
Exposed with AVT	95,219	5001	1.29 (1.25–1.33)	
	=1,030,029			

*Note*: Minimal models implicitly adjusted for matching factors: age, sex, race, ethnicity, site of care, and calendar time. Full models additionally adjusted for: asthma, autoimmune disease, cancer and/or metastases, myocardial infarction, congestive heart failure, peripheral vascular disease, cerebrovascular disease, chronic obstructive pulmonary disease, dementia, diabetes, human immunodeficiency virus, immunosuppression, hypertension, liver disease, Parkinson's disease, rheumatological disease, anticonvulsants, oral corticosteroids, prescription opioids, proton pump inhibitors, statins, selective serotonin reuptake inhibitors, Area Deprivation Index, alcohol status, body mass index, smoking status and Charlson Comorbidity Index.

Abbreviations: AVT, antiviral therapy; VACS, Veterans Aging Cohort Study.

^a^ Calculated from the likelihood ratio test.

Similarly, the association was strongest in the frailest quartile of individuals (p‐interaction = 0.03). The HR for the association between incident HZ and incident fragility fractures for the least frail quartile was 1.11 (95% CI: 1.05, 1.18) compared to 1.25 (95% CI: 1.19, 1.31) in the frailest quartile.

Lastly, there was evidence of effect modification by receipt of AVT. Veterans with HZ who did not receive AVT within 7 days of diagnosis had a 4% (HR = 1.04, 95% CI: 1.01, 1.07) increased risk of fragility fracture compared to Veterans without HZ. Veterans with HZ who did receive AVT within 7 days of diagnosis had a 29% (HR = 1.29, 95% CI: 1.25, 1.33) increased risk of fragility fracture compared to Veterans without HZ.

### 3.4 Sensitivity Analysis

A model restricting exposure ascertainment to 31st December 2019 was consistent with the findings seen in the main model (HR = 1.14, 95% CI: 1.12, 1.17) (Table S4).

The only variable to be excluded from the main analysis due to substantial missingness was the VACS Index (29.3% unexposed and 14.8% exposed). HZ exposure was associated with a 15% (HR = 1.15, 95% CI: 1.13, 1.18) increased risk of fragility fracture when adjusting for the VACS Index, which is consistent with the main model.

## 4 Discussion

Our matched cohort study found that Veterans with HZ had a 15% increased risk of any fragility fracture compared to matched Veterans without HZ after adjusting for socio‐demographic, lifestyle, and clinical confounders. This increased risk was also observed for each fracture site individually and ranged from 8% to 18% increase in risk. The risk of fragility fractures associated with HZ was greater in older Veterans, those with higher levels of frailty, and those who received AVT. Results from sensitivity analyses were consistent with the main findings.

Systemic inflammation is well understood to lead to a state of resorption in bone, and previous evidence has identified the association between many inflammatory conditions and a reduction in bone density [25, 26]. Although symptoms of HZ resolve in 3–5 weeks, there is evidence of continuing immunological reactions and inflammation many years after the reactivation of the VZV [27]. Chronic inflammation leading to a reduction in bone mineral density, which subsequently increases the risk of fragility fractures, presents a plausible mechanism for the association observed in our study. In the short term, acute features of HZ infection, such as neuropathic pain, reduced mobility, fatigue, and delirium, could all increase the likelihood of falls in affected patients. Furthermore, if HZ involves the eye (herpes zoster ophthalmicus) or ear (herpes zoster oticus), then complications such as vision loss or vertigo are also likely to increase the risk of falls. The persistent and potentially debilitating pain of PHN could also present a prolonged risk factor for further falls. Additionally, there is evidence that even short‐term corticosteroid use, as might be involved in the treatment of an immunocompetent patient with HZ, increases the risk of fracture [28].

The findings of our study were consistent with published reports in Taiwan, which observed a 20% increased risk of fragility fracture in HZ diagnosed patients compared to patients without HZ [12]. Similarly to ours, this study also found a higher increased risk of hip (HR = 1.34 vs. HR = 1.08) and vertebral (HR = 1.38 vs. HR = 1.18) fractures in HZ diagnosed patients compared to patients without HZ. The increase in the HR for an association for each fracture type may reflect the lack of adjustment in that study for important risk factors such as alcohol and smoking, which may have more pronounced effects in the exposed population who were found to have a higher comorbidity burden and medication use in their study.

Of the total fractures identified in our study, 40% were vertebral fractures and the HR indicating the highest increased risk was also seen at this fracture site (HR = 1.18). Previous literature identifies vertebral fractures as among the most common osteoporotic fractures with an estimated 1.5 million vertebral compression fractures in the US each year [29, 30]. Despite their incidence, vertebral fractures remain significantly underdiagnosed [31, 32]. Vertebral bodies are composed largely of trabecular bone, which is more susceptible to the effects of early osteoporosis compared to cortical bone [33]. Furthermore, with increasing age, degeneration of the intervertebral discs can lead to altered transmission of load in the spinal column, which can predispose to certain types of vertebral fractures [34]. This is an important finding as vertebral fractures (as well as hip fractures) are both associated with a substantial increased risk in mortality [35].

The relationship observed in the main model was modified by both age and frailty, revealing that the association between HZ and fragility fractures was more pronounced in older and more frail individuals. This is consistent with prior studies that have shown increasing age and frailty as strong independent risk factors for fragility fractures [36, 37]. Using a 3‐level HZ exposure identified that HZ‐exposed individuals who were in receipt of AVT within 7 days had the highest risk of fragility fractures. This finding initially appears unusual as we expected that those who received timely treatment would have a better prognosis compared to HZ‐exposed individuals who did not receive timely treatment. However, it is plausible that this is an example of confounding by indication and individuals with more severe presentations of HZ/comorbidity burden were more likely to receive AVT.

A major strength of our study is the use of a large, national cohort of patients receiving care in the largest integrated healthcare system in the United States, which provides high statistical power to detect an association. The VACS‐National is a rich resource of EHR data and allowed for the thorough adjustment of key socio‐demographic, lifestyle, and clinical confounders to produce an accurate assessment of the association between HZ and fragility fractures. We were able to explore the association between HZ and fragility fractures in a setting with a different healthcare system, health‐seeking behavior, and potentially different patterns of risk factors to those seen in previous literature [12]. The stratified analyses by age, frailty level, and receipt of AVT provided valuable insights into subgroups at heightened risk for fragility fractures. By identifying these high‐risk subgroups, our study contributes to a more nuanced understanding of the relationship between HZ and fracture risk, highlighting a potential need for targeted interventions. Another strength of our study was the use of accurate and validated codes to capture both our exposure and outcome. A validation study of ICD‐9 codes detected HZ with 98% sensitivity and 93% PPV [19]. For incident fragility fracture ascertainment, “overall accuracy of the ICD codes relative to the gold standard (documentation in radiology reports or progress notes) was 98%, and agreement beyond chance (kappa) was substantial (0.94)” [7].

An important limitation is that individuals with more mild presentations of HZ may not access healthcare. Although prior research has indicated that 91% of patients with HZ seek healthcare in the United States, it is unknown whether this health‐seeking behavior is seen in Veterans in VA care [38]. Furthermore, individuals with milder cases of HZ are likely healthier and at lower risk of fragility fractures, introducing potential differential exposure misclassification that may overestimate fracture risk among the comparatively less healthy exposed group. In addition, excluding diagnoses limited to chronic HZ complications only may introduce non‐differential exposure misclassification, likely biasing the effect estimate toward the null. Despite restricting the cohort to patients routinely accessing VA care, potential exposure and outcome misclassification remain for those receiving care outside of the VA. Unrecognized or inaccurately coded cases of HZ may also be misclassified. Additionally, the dataset lacked information on fracture mechanism (e.g., low‐energy trauma), but as misclassification of fragility fractures is likely similar across exposure groups, this would be non‐differential and bias estimates toward the null.

Although extensive adjustment was performed, residual confounding by measured and unmeasured factors remains possible. Key unmeasured variables include lifestyle factors such as physical activity and dietary intake (e.g., vitamin D), which both influence fracture risk [39, 40]. Additionally, we lacked data on parental history of fragility fractures or other genetic susceptibilities, which may contribute to residual confounding. We were unable to update time‐varying covariates such as changes in medication use or the development of new comorbidities during follow‐up. Objective assessment of osteoporosis with dual‐energy x‐ray absorptiometry was unavailable. We were also unable to assess the potential modifying effect of HZ vaccination on fracture risk. A 2022 report by the Government Accountability Office suggested only 30% of eligible adults have received the CDC‐recommended recombinant shingles vaccine despite it being more than 90% effective at preventing shingles [41]. There is evidence that vaccine coverage is even lower among Veterans, with an estimated vaccination rate of 5% in Veterans 50 years or older [42]. Nevertheless, our findings highlight an important potential complication of HZ, underscoring the need to improve vaccination coverage and to prioritize high‐risk individuals for preventive strategies.

Generalizability may be limited given the unique characteristics of our cohort; Veterans are predominantly male (> 93%) and have a higher prevalence of comorbidities than the general population [43, 44]. There is clear evidence of sex disparities in osteoporosis, as osteoporosis is four times more common in females than males [45]. Caution should therefore be exercised when applying these findings to non‐Veteran populations, and future research that focuses on the female population, who face unique fracture risk factors, would be valuable.

In conclusion, healthcare professionals should promote the uptake of the recombinant shingles vaccine to reduce HZ‐related complications. Our findings underscore fragility fractures as a public health concern among male Veterans, a group not typically considered high risk. Future studies should address non‐Veteran and female populations.

## Author Contributions

Study concept and design: Charlotte Warren‐Gash and Christopher T. Rentsch. Acquisition of subjects and/or data: Christopher T. Rentsch. Analysis and interpretation of data: Calif A. A. Yousuf, Charlotte Warren‐Gash, and Christopher T. Rentsch. Preparation of manuscript: Calif A. A. Yousuf, Julie A. Womack, Roger J. Bedimo, Charlotte Warren‐Gash, and Christopher T. Rentsch.

## Disclosure

Sponsor's role: The funders had no role in considering the study design or in the collection, analysis, interpretation of data, writing of the report, or decision to submit the article for publication.

## Conflicts of Interest

The authors declare no conflicts of interest.

## Supporting information


**Figure S1:** Cohort selection process flowchart.
**Figure S2:** DAG of the association between HZ and fragility fractures.
**Table S1:** ICD‐9 and ICD‐10 codes for herpes zoster.
**Table S2:** Validated ICD‐9 and ICD‐10 codes for fragility fracture.
**Table S3:** RECORD Checklist.
**Table S4:** Sensitivity analyses of the association between incident HZ and incident fragility fractures with a restricted exposure ascertainment and including confounders with substantial missingness.

## Data Availability

Due to US Department of Veterans Affairs (VA) regulations and our ethics agreements, the analytic data sets used for this study are not permitted to leave the VA firewall without a data use agreement. This limitation is consistent with other studies based on VA data. However, VA data are made freely available to researchers with an approved VA study protocol. For more information, please visit https://www.virec.research.va.gov or contact the VA Information Resource Center at virec@va.gov.
