# Supplementary material for: Association Between Herpes Zoster and Risk of Incident Fragility Fractures in US Veterans: A Matched Cohort Study
Source: J Am Geriatr Soc. 2025 Oct 17;73(12):3778–88. doi: 10.1111/jgs.70174 (PMC12748036; doi:10.1111/jgs.70174)
Supplement: Supplementary file 1 — Figure S1: Cohort selection process flowchart. Figure S2: DAG of the association between HZ and fragility fractures. Table S1: ICD‐9 and ICD‐10 codes for herpes zoster. Table S2: Validated ICD‐9 and ICD‐10 codes for fragility fracture. Table S3: RECORD Checklist. Table S4: Sensitivity analyses of the association between incident HZ and incident fragility fractures with a restricted exposure ascertainment and including confounders with substantial missingness. [file JGS-73-3778-s001.pdf]

Supplementary material for 'Association between herpes zoster and risk of incident fragility fractures in US veterans: a matched cohort study'

Supplementary Figure S1: Cohort selection process flowchart

Assessed for eligibility
(n = 8,395,080)
-Patients aged ≥40 years with ≥1 year of follow-up during study period

Excluded (n = 220,825)
-Patients with history of HZ (n = 114,277)
-Patients with history of fractures (n = 106,548)

Eligible for matching
(n = 8,201,643)
-Patients with no history of HZ or fractures at cohort entry date

Matched patients
(Analysed in the descriptive analysis, crude fragility fracture incidence rate and minimally adjusted model)
-Matched exposed (n = 229,992)
-Matched unexposed (n = 1,134,519)
229,992 valid matched sets at this stage*

Excluded from the fully adjusted model due to missingness in any of the covariates or not being in a valid match set*
-Exposed patients with missing data (n = 20,759)
-Unexposed patients with missing data (n = 245,422)
-Patients in invalid matched sets after those with missing data were excluded (n = 68,301)

Matched patients
(analysed in the fully adjusted main analysis)
-Matched exposed (n = 207,967)
-Matched unexposed (n = 822,062)
207,967 valid matched sets in the main model*

*A valid matched set was defined as a matched set that included at least one HZ exposed and HZ unexposed patient

Supplementary Table S1: ICD-9 and ICD-10 codes for herpes zoster

**ICD-9 codes for herpes zoster**
053.0, 053.8, 053.9, 053.20, 053.21, 053.22, 053.29, 053.71, 053.79
**ICD-10 codes for herpes zoster**
B02.0, B02.1, B02.30, B02.31, B02.32, B02.33, B02.34, B02.39, B02.7, B02.8, B02.9

Supplementary Table S2: Validated ICD-9 and ICD-10 codes for fragility fracture

**Validated ICD-9 codes for fragility fractures**
Hip<br>820.0X, 820.1X, 820.2X, 820.3X, 820.8, 820.9
Shoulder/upper arm<br>812.0X, 812.1X, 812.2X, 812.3X, 812.4X, 812.5X
Wrist/forearm<br>813.0X, 813.1X, 813.2X, 813.3X, 813.4X, 813.5X813.8X, 813.9X, 814.0X, 814.1X
Vertebral<br>805.2, 805.3, 805.4, 805.5, 805.6, 805.7; 805.8; 805.9
**Validated ICD-10 codes for fragility fractures**
Hip/femoral<br>S72.00%, S72.01%, S72.02%, S72.03%, S72.04%, S72.05%, S72.06%, S72.09%, S72.10%, S72.11%, S72.12%, S72.14%, S72.21%, S72.22%, S72.23%, S72.24%, S72.25%, S72.26%, M80.05%, M80.85%
Shoulder/upper arm<br>M80.02%, M80.82%, S42.20%, S42.21%, S42.22%, S42.23%, S42.24%, S42.25%, S42.26%, S42.29%, S42.30%, S42.32%, S42.33%, S42.34%, S42.35%, S42.36%, S42.39%, S42.40%, S42.41%, S42.42%, S42.45%, S42.46%, S42.47%, S42.49%, S42.90%, S42.91%, S42.92%
Wrist/forearm<br>S52.00%, S52.02%, S52.03%, S52.04%, S52.09%, S52.10%, S52.12%, S52.13%, S52.18%, S52.20%, S52.22%, S52.23%, S52.24%, S52.25%, S52.26%, S52.27%, S52.29%, S52.30%, S52.32%, S52.33%, S52.34%, S52.35%, S52.36%, S52.37%, S52.39%, S52.90%, S52.91%, S52.92%, M80.03%, M80.83%, S52.50%, S52.51%, S52.53%, S52.54%, S52.55%, S52.56%, S52.57%, S52.59%, S52.60%, S52.61%, S52.69%, S62.00%, S62.01%, S62.02%, S62.03%, S62.10%, S62.11%, S62.12%, S62.13%, S62.14%, S62.15%, S62.16%, S62.17%, S62.18%
Vertebral<br>M80.08%, M80.88%, S12.9XX%, S22.00%, S22.01%, S22.02%, S22.03%, S22.04%, S22.05%, S22.06%, S22.07%, S22.08%, S32.00%, S32.01%, S32.02%, S32.03%, S32.04%, S32.05%, S32.10%, S32.11%, S32.12%, S32.13%, S32.14%, S32.15%, S32.16%, S32.17%, S32.19%, S32.2%

Supplementary Figure S2: DAG of the association between HZ and fragility fractures

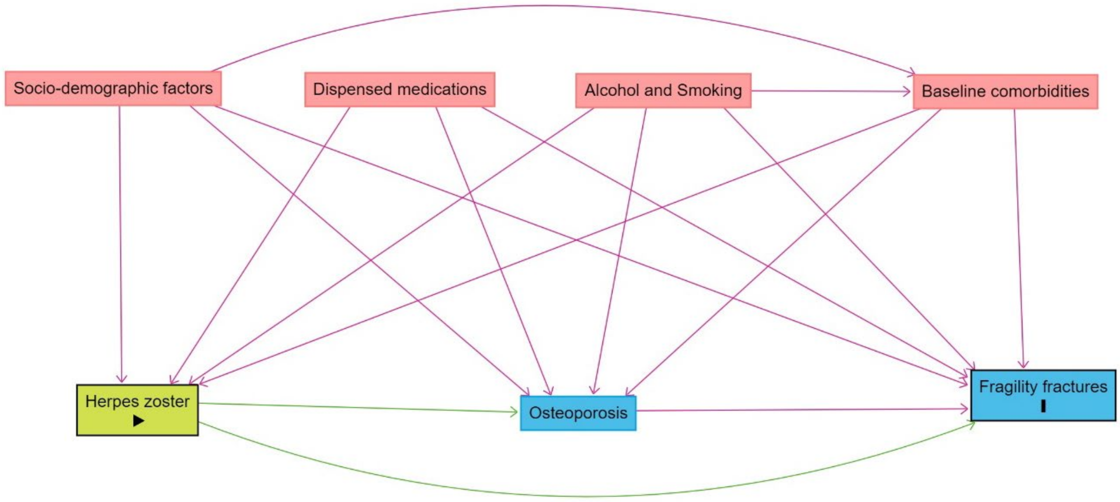

Supplementary Text S1: Covariate definition and handling

Age at index date, sex, race, ethnicity, site of care, and calendar time were used as matching variables as also seen in a previous study [1].

- Age at index date (measured in years) was used to match HZ diagnosed and HZ undiagnosed Veterans who were within 365 days of each other.
- Sex was defined as male or female.
- Race and ethnicity were defined in seven categories: White, Black, Hispanic, Asian, American Indian/Alaska Native, Native Hawaiian/Pacific Islander and Mixed race.
- Site of care refers to which healthcare facility each Veteran attended.
- Matching on calendar time involved matching Veterans with HZ with up to five Veterans who were alive, in care and HZ-free in the same calendar year.

All other covariates included in the study were considered prior to the creation of the dataset as being potential confounders for the relationship between HZ and fragility fractures. We assessed the linearity of continuous variables in our Cox model using martingale residuals. A plot of the residuals against each continuous variable with a fitted line was produced. A random scatter around the line suggested linearity, whereas any systematic deviation indicated non-linearity. If the plot suggested non-linearity of the continuous variable it was included in our model as a categorical variable.

Each covariate in the dataset is described below:

Area Deprivation Index (ADI) is a measure of neighbourhood disadvantage that allows for comparison within the US [2]. A neighbourhood is defined as a census block group which is the smallest geographic area for which the Bureau of the Census collects data [3]. Key domains measured in the ADI include: education, employment, housing-quality, and poverty measures [2]. The ADI is measured quarterly

longitudinally over time with the closest measure to baseline being used in the study. National ADI is measured in percentiles with 1 being the least deprived and 100 being the most deprived. For descriptive analyses we categorised ADI into quintiles with quintile one representing the lowest level of deprivation and quintile five representing the highest level of deprivation. After assessing linearity, ADI was treated as a continuous variable in my main analyses (Supplementary Figure S2).

We combined the Alcohol Use Disorders Identification Test–Consumption (AUDIT-C) questionnaire and ICD-9 and ICD-10 diagnoses for alcohol use disorder (AUD) to form a single alcohol variable that classified patients into 5 mutually exclusive groups as per a previous study [4-7]. AUDIT-C is a three-item questionnaire on the quantity and frequency of alcohol consumption and is routinely collected during annual health visits for all Veterans in care. Abstinent without AUD (AUDIT-C score of 0 and absence of AUD diagnosis), abstinent with AUD (AUDIT-C score of 0 and presence of AUD diagnosis), lower-risk consumption (AUDIT-C score of 1-3 and absence of AUD diagnosis), moderate-risk consumption (AUDIT-C score of 4-7 and absence of AUD diagnosis), and high-risk consumption or AUD (AUDIT-C score of ≥8 or presence of AUD diagnosis with nonzero AUDIT-C score)

Body Mass Index (BMI) was derived from the most recent height and weight measurement prior to baseline. For the descriptive analysis we categorised BMI as: Underweight (<18.5 kg/m^2), Normal weight (18.5kg/m^2 -24.99kg/m^2), Overweight (25.0kg/m^2 -29.99kg/m^2) and Obese (>30kg/m^2). After assessing linearity, BMI was treated as a continuous variable in our main model (Supplementary Figure S2).

Smoking status was identified by the most frequent response in the five years prior to the baseline date. Smoking status categories were: current, former or never.

The VACS Index is a validated and generalisable measure of physiologic frailty that considers factors such as age, organ system injury indicators (haemoglobin, Fibrosis-4 Index, estimated glomerular filtration rate, albumin, white blood cell count) BMI, HIV indicators, and hepatitis C infection [8]. The values of the VACS Index range from 0-100 for most patients with higher values of the VACS Index predicting poorer prognosis and higher mortality risk [7,8]. For both our descriptive analysis and exploration of potential effect modification the VACS Index was categorised into quartiles (1-4, lowest to highest level of frailty).

The Charlson Comorbidity Index (CCI) is a measure of overall comorbidity burden based on clinical diagnosis of comorbid conditions using ICD-9 and ICD-10 codes [5,6]. In our study the values for the CCI ranges from 0-21 with higher scores indicating greater mortality risk and more severe comorbidity [9]. Combining CCI and the VACS Index has been shown to improve prognostic accuracy for mortality for patients in the VA[10]. After assessing linearity, we treated CCI as a continuous variable in our main model (Supplementary Figure S2).

Medication variables were determined from pharmacy dispensing records by searching text strings in patient medical records for the presence of generic or brand names for each medication in the two years prior to baseline. The medications groups included in the study were those that were known to affect bone health

(either risk or protective factors): anticonvulsants, oral corticosteroids, prescription opioids, proton pump inhibitors, statins, and selective serotonin reuptake inhibitors[11-13].

Clinical comorbidities were defined as the presence of one inpatient or two outpatient codes using ICD-9 and ICD-10 codes identified within the two years prior to baseline [5,6]. This however is not the case for immunosuppression, which was identified in the five, two or one year prior to baseline dependent on the condition. The remaining clinical comorbidities were: asthma, autoimmune disease, cancer and/or metastases, myocardial infarction, congestive heart failure, peripheral vascular disease, cerebrovascular disease, chronic obstructive pulmonary disease, dementia, diabetes, human immunodeficiency virus, hypertension, liver disease, Parkinson's disease and rheumatological disease.

Supplementary Text S2: Missing data

The most plausible mechanism for missing data in our study was missing not at random (MNAR). Using BMI as an example, it was considered likely that clinicians are more likely to weigh and measure a patient who is likely to have a BMI outside of the recommended range compared to a patient who appears to have a normal BMI. Therefore, it is plausible that missing data would be more common among those with normal BMI values. Similarly, clinicians may be more likely to record alcohol use in patients who consume alcohol more heavily and thus missing data may be more common among low alcohol consumers. Where missingness has been determined to be MNAR, a complete case analysis is a valid approach[14]. The missing at random assumption necessary for multiple imputation was not considered valid.

In our comparison of incident fragility fractures in HZ exposed and matched controls the characteristics of individuals included in the main analysis (with no missing data) were compared to those who had any level of missingness to assess for a potential selection bias (Supplementary Table S4).

Supplementary Text S3: Additional detail on data management and statistical analysis

The dataset was created by applying the eligibility and inclusion criteria seen in Figure 2 on the VACS-National cohort. The exposure assessment and matching process was then conducted. These steps were performed on VA secure servers.

Biologically implausible values for BMI (<14kg/m^2 or >70kg/m^2) were excluded based on cut-off values used in prior research [15]. Assessing the distribution of BMI found this cutoff to be feasible for my study population which led to 663 patient BMI values being excluded.

A valid matched set was defined as a matched set that included one Veteran with HZ and at least one Veteran without HZ. We excluded all matched sets that were not considered valid prior to undertaking any analysis. After excluding patients with

missing data in the fully adjusted model, we again assessed the validity of all matched sets those that were not considered valid were excluded. The number of matched sets at each stage of the analysis is shown in Figure 2.

We compared the root mean squared error (RMSE) of our fully adjusted and minimally adjusted models to assess for multicollinearity. A comparison of the RMSE in the minimally and fully adjusted models of the primary analysis identified there were no issues with multicollinearity and thus the full model was adopted.

A graphical assessment of the proportional hazard's assumption was undertaken for the main exposure by assessing a lowess smoothed plot of scaled Schoenfeld residuals against time. The lowess smoothed plot of scaled Schoenfeld residuals against time for HZ showed no systematic pattern over time with the smooth curve being approximately horizontal around zero over follow up time. These graphical findings support the validity of the proportional hazard's assumption in the model.

To account for clustering due to cases of an unexposed match later entering the study as an exposed patient we used robust standard errors clustered by patient. As understanding a potential causal effect was of interest, the competing event of death was censored and the analysis focused on the cause specific hazard [16].

There was an a-priori interest in exploring potential effect measure modification by both age and frailty as both are strongly associated with an increased risk of fragility fractures [17]. For this analysis age was categorised into 5 levels: 40-49, 50-59, 60-69, 70-79 and 80+. Frailty was measured by the VACS Index and in this analysis was categorised into quartiles (1-4, lowest to highest risk of mortality). For each analysis, interaction terms were fitted between the potential effect modifier of interest and the primary exposure (HZ) in a fully adjusted model.

Supplementary Table S3: RECORD Checklist

	Item No.	STROBE items	Location in manuscript where items are reported	RECORD items	Location in manuscript where items are reported
Title and abstract					
	1	(a) Indicate the study's design with a commonly used term in the title or the abstract (b) Provide in the abstract an informative and balanced summary of what was done and what was found	(1a) Title<br>(1b) Abstract	RECORD 1.1: The type of data used should be specified in the title or abstract. When possible, the name of the databases used should be included.<br><br>RECORD 1.2: If applicable, the geographic region and timeframe within which the study took place should be reported in the title or abstract.<br><br>RECORD 1.3: If linkage between databases was conducted for the study, this should be clearly stated in the title or abstract.	(1.1) Abstract<br><br>(1.2) Abstract<br><br>(1.3) N/A

Introduction					
Background rationale	2	Explain the scientific background and rationale for the investigation being reported			
Objectives	3	State specific objectives, including any prespecified hypotheses	(3) Introduction		
Methods					
Study Design	4	Present key elements of study design early in the paper	(4) Methods: Study design and population & Figure 1		
Setting	5	Describe the setting, locations, and relevant dates, including periods of recruitment, exposure, follow-up, and data collection	(5) Methods: Study design and population & Methods: Follow up		

Participants	6	*(a) Cohort study* - Give the eligibility criteria, and the sources and methods of selection of participants. Describe methods of follow-up.<br>*Case-control study* - Give the eligibility criteria, and the sources and methods of case ascertainment and control selection. Give the rationale for the choice of cases and controls<br>*Cross-sectional study* - Give the eligibility criteria, and the sources and methods of selection of participants	(6a) Methods: Study design and population & Methods: Follow up & Figure 2: Cohort selection process flowchart.	RECORD 6.1: The methods of study population selection (such as codes or algorithms used to identify subjects) should be listed in detail. If this is not possible, an explanation should be provided.<br>RECORD 6.2: Any validation studies of the codes or algorithms used to select the population should be referenced. If validation was conducted for this study and not published elsewhere, detailed methods and results should be provided.<br>RECORD 6.3: If the study involved linkage of databases, consider use of a flow diagram or other graphical display to demonstrate the data linkage process, including the number of individuals with linked data at each stage.	(6.1) Methods: Study design and population & Methods: Exposure, outcome and covariates & Supplementary Table S1<br><br>(6.2) N/A<br><br>(6.3) N/A

		(b) Cohort study - For matched studies, give matching criteria and number of exposed and unexposed<br>*Case-control study* - For matched studies, give matching criteria and the number of controls per case	(6b) Methods: Study design and population & Table 1: Baseline characteristics by HZ status		
Variables	7	Clearly define all outcomes, exposures, predictors, potential confounders, and effect modifiers. Give diagnostic criteria, if applicable.	(7) Methods: Exposure, outcome and covariates & Supplementary Table S1 & Supplementary Table S2 & Supplementary Text S1	RECORD 7.1: A complete list of codes and algorithms used to classify exposures, outcomes, confounders, and effect modifiers should be provided. If these cannot be reported, an explanation should be provided.	(7.1) Methods: Exposure, outcome and covariates & Supplementary Table S1 & Supplementary Table S2
Data sources/ measurement	8	For each variable of interest, give sources of	(8) Supplementary Text S1		

		data and details of methods of assessment (measurement).<br><br>Describe comparability of assessment methods if there is more than one group			
Bias	9	Describe any efforts to address potential sources of bias	(9) Discussion		
Study size	10	Explain how the study size was arrived at	(10) Figure 2: Cohort selection process flowchart		
Quantitative variables	11	Explain how quantitative variables were handled in the analyses. If applicable, describe which groupings were chosen, and why	(11) Supplementary Text S1		
Statistical methods	12	(a) Describe all statistical methods,	(12a) Methods: Statistical analysis		

		including those used to control for confounding<br>(b) Describe any methods used to examine subgroups and interactions<br>(c) Explain how missing data were addressed<br>(d) *Cohort study* - If applicable, explain how loss to follow-up was addressed<br>(e) Describe any sensitivity analyses	(12b) Methods: Statistical analysis<br><br>(12c) Supplementary Text S3<br><br>(12d) Methods: Follow up & Supplementary Text S2<br><br>(12e) Methods: Sensitivity analysis		

Data access and cleaning methods		..		RECORD 12.1: Authors should describe the extent to which the investigators had access to the database population used to create the study population.<br><br>RECORD 12.2: Authors should provide information on the data cleaning methods used in the study.	(12.1) Supplementary Text S2<br><br>(12.2) Supplementary Text S2
Linkage				RECORD 12.3: State whether the study included person-level, institutional-level, or other data linkage across two or more databases. The methods of linkage and methods of linkage quality evaluation should be provided.	(12.3) N/A
Results					

Participants	13	(a) Report the numbers of individuals at each stage of the study (*e.g.*, numbers potentially eligible, examined for eligibility, confirmed eligible, included in the study, completing follow-up, and analysed)<br>(b) Give reasons for non-participation at each stage.<br>(c) Consider use of a flow diagram	(13a) Figure 2: Cohort selection process flowchart<br><br>(13b) Figure 2: Cohort selection process flowchart<br><br>(13c) Figure 2: Cohort selection process flowchart	RECORD 13.1: Describe in detail the selection of the persons included in the study (*i.e.,* study population selection) including filtering based on data quality, data availability and linkage. The selection of included persons can be described in the text and/or by means of the study flow diagram.	(13.1) Figure 2: Cohort selection process flowchart
Descriptive data	14	(a) Give characteristics of study participants (*e.g.*, demographic, clinical, social) and information on exposures and potential confounders<br>(b) Indicate the number of participants with	(14a) Results: Descriptive analysis + Table 1: Baseline characteristics by HZ status		

		missing data for each variable of interest<br>(c) *Cohort study* - summarise follow-up time (*e.g.*, average and total amount)	(14b) Table 1: Baseline characteristics by HZ status<br><br>(14c) Table 1: Baseline characteristics by HZ status		
Outcome data	15	*Cohort study* - Report numbers of outcome events or summary measures over time<br>*Case-control study* - Report numbers in each exposure category, or summary measures of exposure<br>*Cross-sectional study* - Report numbers of outcome events or summary measures	(15) Table 2: Association between incident HZ and incident fragility fractures		

Main results	16	(a) Give unadjusted estimates and, if applicable, confounder-adjusted estimates and their precision (e.g., 95% confidence interval). Make clear which confounders were adjusted for and why they were included<br>(b) Report category boundaries when continuous variables were categorized<br>(c) If relevant, consider translating estimates of relative risk into absolute risk for a meaningful time period	(16a) Results: Comparison of incident fragility fractures in HZ exposed and matched controls & Table 2: Association between incident HZ and incident fragility fractures<br>(16b) Results: Comparison of incident fragility fractures in HZ exposed and matched controls & Table 2: Association between incident HZ and incident fragility fractures<br>(16c) N/A		

Other analyses	17	Report other analyses done—e.g., analyses of subgroups and interactions, and sensitivity analyses	(17) Results: Comparison of incident fragility fractures in HZ exposed and matched controls & Results: Sensitivity analyses & Table 3: Association between incident HZ and incident fragility fractures stratified by age group, physiologic frailty and receipt of AVT & Supplementary Table S5: Sensitivity analyses of the association between incident HZ and incident fragility fractures		

			with a restricted exposure ascertainment and including confounders with substantial missingness		
Discussion					
Key results	18	Summarise key results with reference to study objectives	(18) Discussion		
Limitations	19	Discuss limitations of the study, taking into account sources of potential bias or imprecision. Discuss both direction and magnitude of any potential bias	(19) Discussion	RECORD 19.1: Discuss the implications of using data that were not created or collected to answer the specific research question(s). Include discussion of misclassification bias, unmeasured confounding, missing data, and changing eligibility over time, as they pertain to the study being reported.	(19) Discussion
Interpretation	20	Give a cautious overall interpretation of results	(20) Discussion		

		considering objectives, limitations, multiplicity of analyses, results from similar studies, and other relevant evidence			
Generalisability	21	Discuss the generalisability (external validity) of the study results	(20) Discussion		
Other Information					
Funding	22	Give the source of funding and the role of the funders for the present study and, if applicable, for the original study on which the present article is based	(22) Important disclosures		
Accessibility of protocol, raw data, and				RECORD 22.1: Authors should provide information on how to access any supplemental information such as	(22.1) Supporting information

programming code				the study protocol, raw data, or programming code.	

Supplementary Text S4: Additional results

The median time to hip/femoral fracture overall was 3.8 years (IQR:1.8,6.5) for Veterans without HZ and 3.9 years (IQR:1.8,6.7) for Veterans with HZ. The median time to shoulder/upper arm fracture overall was 3.7 years (IQR:1.7,6.5) for Veterans without HZ and 3.8 years (IQR:1.6,6.8) for Veterans with HZ. The median time to vertebral fracture overall was 4.5 years (IQR:2.1,7.2) for Veterans without HZ and 4.3 years (IQR:1.9,7.3) for Veterans with HZ. Lastly, the median time to wrist/forearm fracture overall was 3.5 years (IQR:1.6,6.3) for Veterans without HZ and 3.6 years (IQR:1.6,6.4) for Veterans with HZ.

The distribution of characteristics among Veterans with no missing data (those included in the primary analysis) versus Veterans with any missing data (those excluded from the primary analysis) were compared (Supplementary Table S4). The distribution of age, sex, race and ethnicity were comparable between groups. However, those with missing data were more likely to have entered the study earlier than those without missing data (baseline year 2008-2011: 41.5% vs 29.3%). Veterans without missing data were more likely to have a higher comorbidity burden (as measured by the CCI) and be prescribed any of the 6 medications included in the study.

Supplementary Table S4: Sensitivity analyses of the association between incident HZ and incident fragility fractures with a restricted exposure ascertainment and including confounders with substantial missingness

Herpes zoster diagnosis	N of patients	N of fragility fracture	Fully adjusted model HR (95% CI)
**Restricted exposure ascertainment**			
**Undiagnosed**	728,370	26,444	1 (reference)
**Diagnosed**	185,012	9,421	1.14 (1.12-1.17)
	=913,382		
**Including the VACS Index**			
**Undiagnosed**	675,982	23,196	1 (reference)
**Diagnosed**	180,134	8,611	1.15 (1.13-1.18)
	=856,116		

Abbreviations: VACS, Veterans Aging Cohort Study.
*Calculated from the likelihood ratio test
Minimal models implicitly adjusted for matching factors: age, sex, race, ethnicity, site of care, and calendar time. Full models additionally adjusted for: asthma, autoimmune disease, cancer and/or metastases, myocardial infarction, congestive heart failure, peripheral vascular disease, cerebrovascular disease, chronic obstructive pulmonary disease, dementia, diabetes, human immunodeficiency virus, immunosuppression hypertension, liver disease, Parkinson's disease, rheumatological disease, anticonvulsants, oral corticosteroids, prescription opioids, proton pump inhibitors, statins, selective serotonin reuptake inhibitors, Area Deprivation Index, alcohol status, body mass index, smoking status and Charlson Comorbidity Index.

Supplementary References

1. Tunnicliffe L, Weil RS, Breuer J, et al. Herpes Zoster and Risk of Incident Parkinson's Disease in US Veterans: A Matched Cohort Study. *Movement Disorders*. 2024;39(2):438-444. doi:https://doi.org/10.1002/mds.29701
2. Kind AJH, Buckingham WR. Making Neighborhood-Disadvantage Metrics Accessible - The Neighborhood Atlas. *N Engl J Med*. Jun 28 2018;378(26):2456-2458. doi:10.1056/NEJMp1802313
3. Bureau TUSC. What are census blocks? https://www.census.gov/newsroom/blogs/random-samplings/2011/07/what-are-census-blocks.html
4. Bush K, Kivlahan DR, McDonell MB, Fihn SD, Bradley KA. The AUDIT alcohol consumption questions (AUDIT-C): an effective brief screening test for problem drinking. Ambulatory Care Quality Improvement Project (ACQUIP). Alcohol Use Disorders Identification Test. *Arch Intern Med*. Sep 14 1998;158(16):1789-95. doi:10.1001/archinte.158.16.1789
5. Organization WH. *International classification of diseases:[9th] ninth revision*. World Health Organization; 1978.
6. World Health O. ICD-10 : international statistical classification of diseases and related health problems : tenth revision. 2nd ed. Geneva: World Health Organization; 2004.
7. Cartwright EJ, Pierret C, Minassian C, et al. Alcohol Use and Sustained Virologic Response to Hepatitis C Virus Direct-Acting Antiviral Therapy. *JAMA Network Open*. 2023;6(9):e2335715-e2335715. doi:10.1001/jamanetworkopen.2023.35715
8. Ambia J, Ingle SM, McGinnis K, et al. Discrimination of the Veterans Aging Cohort Study Index 2.0 for Predicting Cause-specific Mortality Among Persons With HIV in Europe and North America. *Open Forum Infectious Diseases*. 2024;11(7)doi:10.1093/ofid/ofae333
9. Charlson ME, Carrozzino D, Guidi J, Patierno C. Charlson Comorbidity Index: A Critical Review of Clinimetric Properties. *Psychother Psychosom*. 2022;91(1):8-35. doi:10.1159/000521288
10. McGinnis KA, Justice AC, Marconi VC, et al. Combining Charlson comorbidity and VACS indices improves prognostic accuracy for all-cause mortality for patients with and without HIV in the Veterans Health Administration. *Front Med (Lausanne)*. 2023;10:1342466. doi:10.3389/fmed.2023.1342466
11. Panday K, Gona A, Humphrey MB. Medication-induced osteoporosis: screening and treatment strategies. *Ther Adv Musculoskelet Dis*. Oct 2014;6(5):185-202. doi:10.1177/1759720x14546350
12. Lin TK, Chou P, Lin CH, Hung YJ, Jong GP. Long-term effect of statins on the risk of new-onset osteoporosis: A nationwide population-based cohort study. *PLoS One*. 2018;13(5):e0196713. doi:10.1371/journal.pone.0196713
13. Saunders KW, Dunn KM, Merrill JO, et al. Relationship of opioid use and dosage levels to fractures in older chronic pain patients. *J Gen Intern Med*. Apr 2010;25(4):310-5. doi:10.1007/s11606-009-1218-z
14. Hughes RA, Heron J, Sterne JAC, Tilling K. Accounting for missing data in statistical analyses: multiple imputation is not always the answer. *International Journal of Epidemiology*. 2019;48(4):1294-1304. doi:10.1093/ije/dyz032

15. Booth HP, Prevost AT, Gulliford MC. Epidemiology of clinical body mass index recording in an obese population in primary care: a cohort study. *J Public Health (Oxf)*. Mar 2013;35(1):67-74. doi:10.1093/pubmed/fds063
16. Pintilie M. Analysing and interpreting competing risk data. *Stat Med*. Mar 15 2007;26(6):1360-7. doi:10.1002/sim.2655
17. Bouvard B, Annweiler C, Legrand E. Osteoporosis in older adults. *Joint Bone Spine*. 2021/05/01/ 2021;88(3):105135. doi:https://doi.org/10.1016/j.jbspin.2021.105135
